# Evidence of previous SARS-CoV-2 infection in seronegative patients with long COVID

**DOI:** 10.1016/j.ebiom.2022.104129

**Published:** 2022-06-27

**Authors:** Benjamin A. Krishna, Eleanor Y. Lim, Lenette Mactavous, NIHR BioResource Team, Paul A. Lyons, Rainer Doffinger, John R. Bradley, Kenneth G.C. Smith, John Sinclair, Nicholas J. Matheson, Paul J. Lehner, Mark R. Wills, Nyaradzai Sithole

**Affiliations:** a Cambridge Institute of Therapeutic Immunology & Infectious Disease (CITIID), Cambridge CB2 0AW, UK; b Department of Medicine, University of Cambridge, Cambridge CB2 0QQ, UK; c Department of Infectious Diseases, Cambridge University Hospitals NHS Foundation Trust, Cambridge CB2 0QQ, UK; d Department of Clinical Biochemistry and Immunology, Cambridge University Hospitals NHS Foundation Trust, Cambridge CB2 0QQ, UK; e National Institute for Health Research (NIHR) Cambridge Biomedical Research Centre, Cambridge CB2 0QQ, UK; f Cambridge NIHR BioResource Centre, Cambridge University Hospitals NHS Foundation Trust, Cambridge CB2 0QQ, UK; g Department of Renal Medicine, Cambridge University Hospitals NHS Foundation Trust, Cambridge CB2 0QQ, UK; h NHS Blood and Transplant, Cambridge CB2 0PT, UK

**Keywords:** Long COVID, T cell, Assay, SARS-CoV-2, Immunity

## Abstract

**Background:**

There is currently no consensus on the diagnosis, definition, symptoms, or duration of COVID-19 illness. The diagnostic complexity of Long COVID is compounded in many patients who were or might have been infected with SARS-CoV-2 but not tested during the acute illness and/or are SARS-CoV-2 antibody negative.

**Methods:**

Given the diagnostic conundrum of Long COVID, we set out to investigate SARS-CoV-2-specific T cell responses in patients with confirmed SARS-CoV-2 infection and/or Long COVID from a cohort of mostly non-hospitalised patients.

**Findings:**

We discovered that IL-2 release (but not IFN-γ release) from T cells in response to SARS-CoV-2 peptides is both sensitive (75% +/−13%) and specific (88%+/−7%) for previous SARS-CoV-2 infection >6 months after a positive PCR test. We identified that 42–53% of patients with Long COVID, but without detectable SARS-CoV-2 antibodies, nonetheless have detectable SARS-CoV-2 specific T cell responses.

**Interpretation:**

Our study reveals evidence (detectable T cell mediated IL-2 release) of previous SARS-CoV-2 infection in seronegative patients with Long COVID.

**Funding:**

This work was funded by the Addenbrooke's Charitable Trust (900276 to NS), NIHR award (G112259 to NS) and supported by the NIHR Cambridge Biomedical Research Centre. NJM is supported by the MRC (TSF MR/T032413/1) and NHSBT (WPA15-02). PJL is supported by the Wellcome Trust (PRF 210688/Z/18/Z, 084957/Z/08/Z), a Medical Research Council research grant MR/V011561/1 and the United Kingdom Research and a Innovation COVID Immunology Consortium grant (MR/V028448/1).

**Research in context**

**Evidence before this study**

Diagnosing Long COVID is a difficult task for clinicians as there is currently no test to distinguish Long COVID from diseases with similar symptoms such as other post viral syndromes and chronic fatigue. Among other factors, it is important to establish whether patients have been infected with SARS-CoV-2. This was a problem for the Long COVID clinic at Addenbrooke's hospital, as most patients with Long COVID symptoms were infected from March to May 2020, before widespread testing in the UK. Memory T cells are generated after SARS-CoV-2 infection, and these cells respond to stimulation with peptides from SARS-CoV-2. We endeavoured to use this response to retrospectively diagnose patients as having had COVID-19. Although blood antibody tests have been used to achieve this, antibodies are known to wane after infection and vaccination means that everyone has antibodies against spike protein.

**Added value of this study**

We used highly sensitive fluorospot assays on peripheral blood mononuclear cells from patients who recovered from confirmed COVID-19, compared to samples collected from before the pandemic. We found that stimulating patient samples with peptides from nucleocapsid and membrane proteins caused interleukin-2 production. By measuring interleukin-2 production, we could distinguish between those who had been infected from those who had not. We then applied this same approach to our cohort with Long COVID, allowing us to identify antibody seronegative patients who had clear T cell responses, indicating previous infection.

**Implications of all the available evidence**

This study demonstrates that T cell assays are a sensitive and effective method to determine past SARS-CoV-2 infection. This could benefit patients with Long COVID by confirming their belief that they had COVID-19 and allow clinicians to diagnose Long COVID based on symptom profile and evidence of past infection.

Alt-text: Unlabelled box

## Introduction

Since the initial reports of SARS-CoV-2 infection in December 2019, hundreds of millions of people have been infected, most of whom experience an asymptomatic or mild acute infection, including around 95% of those in the UK.^1^ However, follow up studies suggest that 0.2–30% of patients experience a plethora of persistent symptoms, variously termed ‘Long COVID’, ‘post-acute sequelae of COVID-19’ (PASC) or post-COVID syndrome.^2^, ^3^, ^4^ This large variation is due to methodological differences between studies. Studies which find high rates of Long COVID focus on earlier time points after disease,^5^ study hospitalised patients exclusively,^6^ or use patient-reported data rather than health records.^2^^,^^4^ Long COVID risk increases with age and is higher in women.^5^ Long COVID is consistent with previous coronavirus outbreaks where at least 10% of individuals infected with MERS (Middle east respiratory syndrome) or SARS-CoV-1^7^, ^8^, ^9^ experienced prolonged symptoms. However, unlike MERS and SARS-CoV-1 sequelae, there are reports of Long COVID affecting individuals after only mild illness.^10^ The remitting and relapsing nature of the illness, lack of consensus regarding the definition for Long COVID, heterogeneity of the disease and lack of biomarker/s makes the diagnosis of Long COVID challenging, with patients reporting a wide range of symptoms including fatigue, fever, headache, dyspnoea, and anosmia.^5^^,^^11^^,^^12^

Diagnosis of SARS-CoV-2 infection using RT-qPCR detection of viral genome can have sensitivity as low as 70%,^13^ with an estimated mean sensitivity of 89%.^14^ Ideally the test has to be performed during peak viral load, beyond which the rate of false negatives increases.^15^ False negative rates are higher in women and younger people,^16^ but can also be caused by laboratory errors, clerical errors or inadequate swabbing technique.^17^ On top of this, testing has not always been easily accessible: from January to May 2020 in the UK, RT-qPCR nasopharyngeal swab testing was not available in the community; during the omicron wave in December 2021, supply of tests could not match demand, and the costs of testing restricts uptake for some people. This means that a significant number of non-hospitalised patients present with symptoms consistent with Long COVID but do not have a positive RT-qPCR result. This is a significant diagnostic challenge, as there is no objective record of past infection with SARS-CoV-2.^18^ Serological assays are useful but underestimate the prevalence of infection and immunity status to SARS-CoV-2 as blood IgG, which can be measured by binding or neutralising assays, against spike or nucleocapsid wanes over time; with mild illness, rapid viral clearance and low antibody response following acute infection, being the most common reasons attributed to failure to detect antibodies.^19^, ^20^, ^21^, ^22^, ^23^, ^24^, ^25^ Furthermore, mass vaccination has induced anti-spike IgG in most individuals, rendering anti-spike IgG unreliable for retrospective diagnosis of SARS-CoV-2. A more specific test would help to rule out false positives: more common in rapid antigen testing^26^ but also caused by contamination and incorrectly determining the cut off Ct value for equivocal results.^27^ False positives could also cause diagnostic issues by triaging patients experiencing fatigue into Long COVID treatments, when their symptoms are caused by another factor.

Antigen-specific memory T cell responses are clinically useful for retrospective diagnosis of infection.^28^ Interleukin-2 (IL-2) and Interferon (IFN-γ) CD4^+^ and CD8^+^ T cell responses specific to SARS-CoV-2 spike (S), nucleocapsid (N) and membrane (M) peptides are detected in PCR-positive individuals up to 12 months after infection, including in those who are seronegative.^20^^,^^29^, ^30^, ^31^, ^32^, ^33^

Given the limitations of serological assays for retrospective diagnosis of SARS-CoV-2, we decided to use a robust SARS-CoV-2 T cell based Fluorospot assay to measure IL-2 and IFN-γ after stimulation with spike, nucleocapsid, and membrane peptides which to our knowledge has not been applied in the context of Long COVID patients. Although other studies have used IFN-γ for SARS-CoV-2 based T cell assays,^34^ we found that IL-2 was more sensitive than IFN-γ for discriminating between a cohort of patients with RT-qPCR confirmed SARS-CoV-2 compared to an unexposed control group.

## Methods

### Ethics

The Long COVID study patients were recruited and consented under the Cambridge COVID-19 NIHR BioResource joint Consent Form (Research Ethics Committee (NRES number (REC)) no. T1gC1) study NBR87. Informed consent was obtained from all participants for the rest of the study.

### Sample collection

Study participants were recruited between 31^st^ of May 2020 and 31^st^ of July 2021 from patients attending the Infectious Diseases led Long COVID clinic at Addenbrooke's Hospital. The majority were non-hospitalised patients from the initial phase of the pandemic and the clinical and epidemiological history played the most significant part in triaging patients into the Long COVID clinic. However, a combination of any of the following parameters were used to triage patients into the clinic; epidemiological and clinical history (both initially assessed by referring General Practitioners), a confirmed diagnosis of COVID-19 by nucleic acid amplification test (including point-of-care testing) and SARS-CoV-2 seropositivity.

The COVID confirmed hospitalised patients (Day 28, Day 90 & Day 180), were enrolled following admission to Addenbrooke's hospital, Royal Papworth and Cambridge and Peterborough Foundation Trust with a confirmed diagnosis of COVID-19 via a positive RT-qPCR test for SARS-CoV-2 as stated in.^35^ Recruitment of inpatients at Addenbrooke's Hospital and health-care workers was undertaken by the National Institute for Health Research (NIHR) Cambridge Clinical Research Facility outreach team and the NIHR BioResource research nurse team as stated in.^35^ Each participant provided 32ml of peripheral venous blood collected into a 9-ml sodium citrate tube. Clinical data was collected at clinic visit and routine laboratory tests and inflammatory cytokine panel were assayed appropriately where clinically relevant.

### Serology testing

SARS-CoV-2 serology was measured using an UKAS accredited COVID-19 serology test, using multiplex particle-based flow cytometry (Luminex): Recombinant SARS-CoV-2 N, S and RBD were covalently coupled to distinct carboxylated bead sets (Luminex; Netherlands) to form a 3-plex assay. The S protein construct used is S-R/PP.^36^ The RBD protein construct used is described by Stadlbauer et al.^37^ Beads were first activated with 1-ethyl-3-[3-dimethylaminopropyl]carbodiimide hydrochloride (Thermo Fisher Scientific) in the presence of N-hydroxysuccinimide (Thermo Fisher Scientific), according to the manufacturer's instructions, to form amine-reactive intermediates. The activated bead sets were incubated with the corresponding proteins at a concentration of 50 μg/ml in the reaction mixture for 3 hours at room temperature on a rotator. Beads were washed and stored in a blocking buffer (10 mM PBS, 1% BSA, 0.05% NaN3).

The N-, S- and RBD-coupled bead sets were incubated with proband sera at a 1/100 dilution for 1 h in 96-well filter plates (MultiScreen HTS; Millipore) at room temperature in the dark on a horizontal shaker. Fluids were aspirated with a vacuum manifold and beads were washed three times with 10 mM PBS/0.05% Tween 20. Beads were incubated for 30 min with a PE-labelled anti-human IgG-Fc antibody (Leinco/Biotrend RRID:AB_2892928), washed as described above, and resuspended in 100 μl PBS/Tween. They were then analysed on a Luminex analyser (Luminex/R&D Systems RRID:SCR_018025) using Exponent Software V31. Specific binding was reported as mean fluorescence intensities (MFI). N protein was kindly provided by Dr Leo James. RBD was provided by Dr James Nathan. Trimeric S was provided by Dr John Briggs.

### PBMC isolation from patient blood

Peripheral blood mononuclear cells (PBMCs) were isolated from citrated blood samples by layering blood onto Lymphoprep (Axis-shield, Oslo, Norway) and performing density gradient centrifugation at 1200 xg for 10 min. PBMCs at the interface were collected and washed 2x in PBS.

### Dual fluorospot assays

We used peptide pools as recently published^38^: “A peptide pool was generated using the following: 1. PepTivator SARS-CoV-2 Prot_S containing the sequence domains aa 304–338, 421–475, 492–519, 683–707, 741–770, 785–802, and 885–1273 and S1 N-terminal S1 domain of the surface glycoprotein (“S”) of SARS-Coronavirus 2 (GenBank MN908947.3, Protein QHD43416.1). 2. The PepTivator SARS-CoV-2 Prot_S1 containing the aa sequence 1–692. The peptides used are 15aa amino acids with 11 amino acid overlaps.” In addition to these, we also used PepTivator SARS-CoV-2 Prot_N covering the entire sequence of Nc (GenBank MN908947.3, Protein QHD43423.2) and PepTivator SARS-CoV-2 Prot_M covering the entire sequence of membrane (GenBank MN908947.3, Protein QHD43419.1).

2 × 10^5^ PBMCs suspended in TexMACS (Miltenyi Biotech) supplemented with 5% Human AB serum (Sigma Aldrich) were incubated on FluoroSpot plates coated with Human IFN-γ and IL-2 antibodies [FluoroSpot (Mabtech AB, Nacka Strand, Sweden)] in duplicate with open reading frame (ORF) mix peptides (final peptide concentration 2 μg/ml/peptide) as well as a TexMACS-only negative control and positive control mix [containing anti-CD3 (Mabtech AB; **RRID:AB_907218**), Staphylococcus Enterotoxin B, and Lipopolysaccharide (all Sigma-Aldrich)] at 37 °C in a humidified CO2 atmosphere for 48 h. The cells and medium were decanted from the plate and the assay developed following the manufacturer's instructions. Developed plates were read using an AID iSpot reader (Oxford Biosystems, Oxford, UK) and counted using AID EliSpot v7 software (Autoimmun Diagnostika GmbH, Strasberg, Germany) using distinct counting protocols for IFN-γ and IL-2 secretion. Donor results were discounted from further analysis if there was less than 100 sfu in the positive control relative to the background sfu. All data were then corrected for background cytokine production.

### Sequence analysis

Sequences for S/N/M from SARS-CoV-2, SARS-CoV-1, OC43, HKU1, NL63 and 229E (GenBank numbers: QHD43416.1, NP_828851.1, YP_009555241.1, AGW27872.1, APF29063.1, NP_073551.1; QHD43423.2, AYV99827.1, AXX83383.1, ABG77571.1, ABI20791.1, AAA45463.1; QHD43419.1, ACZ71786.1, AAA45462.1, AGW27884.1, ABD34826.1, AAA45461.1) were aligned using ClustalX software.^39^ Sequence alignments were visualised using UGene software^40^ coloured to indicate areas of high percentage identity.

### Statistics

Data were determined to be non-parametric by Shapiro-Wilk analysis. We therefore used non-parametric statistical analysis throughout. Kruskal-Wallis one-way analysis of variance (ANOVA) was used for three way comparisons with Dunn's multiple comparisons test. Wilcoxon signed-rank tests were used for comparisons of antibody levels in the same patients before and after vaccine (paired two group comparisons).

For fluorospot assays, each condition (each donor and each peptide stimulation) was run in duplicate. The average number of spots for the negative control (no peptide stimulation) was subtracted from each condition (spike, nucleocapsid, membrane, CEF and anti-CD3 stimulated cells), to account for background IL-2 and IFN-γ production and calculate the specific response to peptide stimulation. The number of spots was then divided by total cells loaded, to account for differences in cell number. Data was graphed as spot forming units (SFU) per 10^6^ cells. Values at or below zero were plotted as 0.1 to allow their visualisation on logarithmic axes. Donor samples were not randomised or blinded.

Sample size was computed using a two-sample t-test power calculation based on preliminary data, with an estimated difference in the means of 51 and assumed common standard deviation of 55. which suggested that based on the difference between the means of two independent groups (unexposed vs D180 infected), that to achieve a type 1 error rate of 0.01 and power 90%, we would require 35 members in each group; which was surpassed.

### Role of the funding source

The funders had no role in the design, execution, or analysis of the study.

## Results

### IL-2 release predicts previous SARS-CoV-2 infection in patients with Long COVID

From May 2020 to July 2021, we recruited 72 patients who attended the Infectious Diseases-led Long COVID clinic at Cambridge University Hospital (Addenbrooke's). These patients were referred to the clinic on the basis that they reported symptoms consistent with Long COVID, which lasted more than 6 months and which significantly reduced their ability to function on the daily basis. The objective was to set up a T-cell based assay to determine evidence of previous SARS-CoV-2 infection in patients with symptoms in keeping with Long COVID but were seronegative for both anti-Spike and anti-Nucleocapsid IgG. In addition to the research bloods, routine clinical bloods, demographic, and clinical data were collected. Of the 72 patients, 83% (60/72) were non-hospitalised and 17% (12/72) were hospitalised (Table 1) at the time of their initial illness. The median age of the patients was 46.5 years (interquartile range (IQR) 35–58 years) with 61% (44/72) female. Only 24% (17/72) were SARS-CoV-2 antibody positive for both anti-Spike and anti-Nucleocapsid, whereas 76% (55/72) were seronegative for both. None of the patients were positive for anti-Spike but not anti-Nucleocapsid or vice versa, which is commonly found in immunocompetent patients using our assay. We did not find any correlation between seropositivity and age, sex, symptom severity, or time since symptom onset. 50% of hospitalised patients were seropositive vs 25% of non-hospitalised patients, which did not reach statistical significance likely because only 12 patients in the Long COVID cohort were hospitalised. Sixty-two and half percent had comorbidities, of which the most frequent was asthma and/or COPD (Table 1).

**Table 1:** Demographic and baseline characteristics of the undifferentiated Long COVID patients.

*N*	72
Age, median (IQR)	46.5 (35–58)
18–30 years	10/72 (14%)
31–45 years	25/72 (35%)
46–60 years	20/72 (28%)
>60 years	17/72 (24%)
% male	28/72 (39%)
%PCR positive	7/72 (9.7%)
% seropositive (both anti-S and anti-N)	21/72 (29%)
% seronegative	51/72 (71%)
%hospitalised	12/72 (17%)
%mild illness	44/72 (61%)
Comorbidities	45/72 (63%)
Hypertension	9/72 (12.5%)
Diabetes Mellitus	4/72 (5.5%)
COPD, Asthma	19/72 (26%)
Anxiety/Depression	7/72 10%)
Cancer/Immunosuppression	3/72 (4%)
Obesity	1/72 (1%)
Chronic Heart Disease	3/72 (4%)
Time since symptoms (months +/- SD)	6.97 +/−2.83

Of the 72 patients recruited, the salient symptoms associated with Long COVID were: fatigue (44%, 32/72), shortness of breath (8.3%, 6/72), brain fog/memory/concentration problems (1.4%, 1/72), chest pains (6.9%, 5/72), palpitations (8.3%, 6/72) and only (2.8%, 2/72) had persistent fever (Table 2).

**Table 2:** Main symptoms at presentation of the Long COVID patients.

Symptoms	All	18–30yrs	31–45yrs	46–60yrs	>60yrs	IL-2 positive	IL-2 negative
Fatigue	32	5	9	13	5	12	20
Shortness of breath	6	1	3	1	1	2	4
Brain fog/memory/concentration problems	1	0	0	1	0	0	1
Peripheral numbness	1	0	1	0	0	1	0
Chest pains	5	1	3	1	0	2	3
Palpitations	6	2	2	2	0	3	3
Fever	2	0	0	2	0	0	2
Altered taste/smell	2	0	1	1	0	2	0

To address our objective, we set up a highly sensitive, dual-colour cytokine (IFN-γ and IL-2) T cell FluoroSpot assay to measure and characterise SARS-CoV-2 specific T cell responses to a pool of peptides generated according to the predicted amino acid sequence of Spike (S), Nucleocapsid (N) and Membrane (M) proteins of the original Wuhan SARS-CoV-2 strain. As our cohorts were infected between April 2020 and October 2020, most were very likely infected with Wuhan SARS-CoV-2 strains. We analysed IL-2 and IFN-γ release from peripheral blood mononuclear cells (PBMCs) collected from the cohort of Long COVID patients.

For positive controls, we used PBMC samples from a cohort of patients with RT-qPCR proven SARS-CoV-2 infection (covering asymptomatic to severe disease). This group was stratified into samples taken 28 days (D28) and from the same patients 90 days (D90) and 180 days (D180) post diagnosis. This allowed us to assess the durability of IL-2 and IFN-γ specific T-cell responses over time. We used PBMC samples from unexposed healthy blood donors collected between 2014 and 2018 as negative controls.

**Figure 1:**
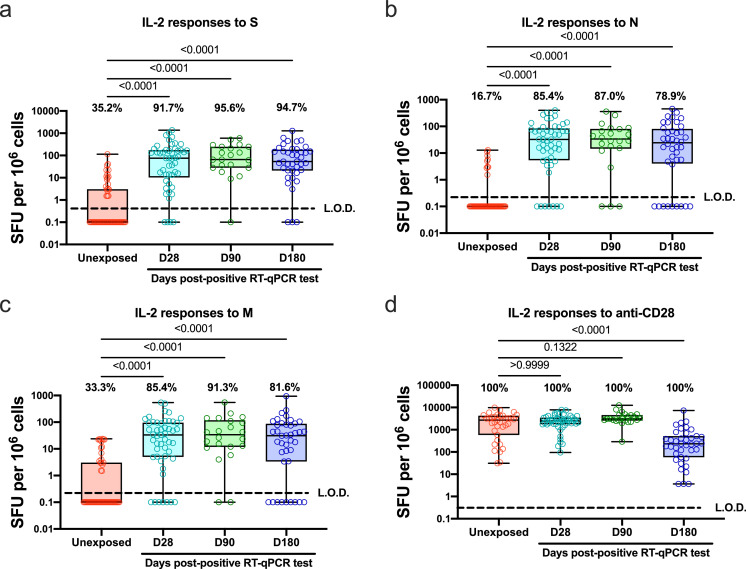
**Donors with confirmed SARS-CoV-2 infection show increased IL-2 responses to Spike, nucleocapsid and membrane peptides**. PBMCs were isolated from negative control unexposed donors (red), positive-control RT-qPCR-confirmed donors at 28, 90 or 180 days post PCR test (cyan, green, blue). These PBMCs were stimulated with spike (A), nucleocapsid (B) or membrane (C) peptides or anti-CD28 as a positive control (d). IL-2 responses were measured by fluorospot assay as spot forming units per million PBMCs. Each condition was run in duplicate and the number of spots quantified by a peptide-negative, unstimulated control was subtracted to remove background cytokine production. Zero results are set as 0.1 to allow their inclusion on a log scale. L.O.D. = limit of detection. Significance calculated by Kruskal-Wallis ANOVA, with Dunn's multiple comparison test between unexposed and each infected group. (For interpretation of the references to color in this figure legend, the reader is referred to the web version of this article.)

**Figure 2:**
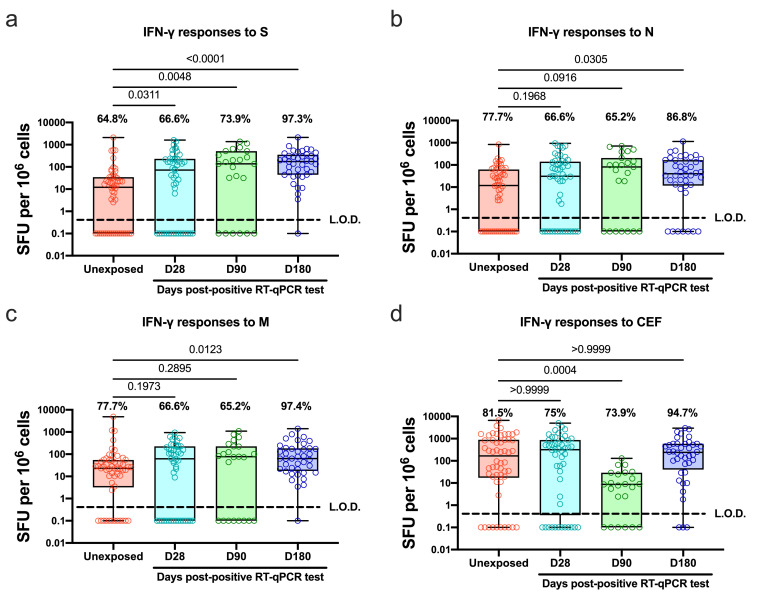
**Donors with confirmed SARS-CoV-2 infection show inconsistently higher IFN-γ responses to Spike, nucleocapsid and membrane peptides**. PBMCs were isolated from negative control unexposed donors (red), positive-control RT-qPCR-confirmed donors at 28, 90 or 180 days post PCR test (cyan, green, blue). These PBMCs were stimulated with spike (A), nucleocapsid (B) membrane (C), or positive-control cytomegalovirus/flu/EBV (CEF, d) peptides. IFN-γ responses were measured by fluorospot assay as spot forming units per million PBMCs. Each condition was run in duplicate and an unstimulated control was subtracted to remove background cytokine production. Zero results are set as 0.1 to allow their inclusion on a log scale. L.O.D. = limit of detection. Significance calculated by Kruskal-Wallis ANOVA, with Dunn's multiple comparison test between unexposed and each infected group. (For interpretation of the references to color in this figure legend, the reader is referred to the web version of this article.)

Median IL-2 responses were significantly higher (78.9–95.6% responding) for all three SARS-CoV-2 peptide pools in our confirmed positive cohort at D28, D90, and D180 (Figure 1a-d) relative to our unexposed negative control cohort. The overall percentage of the cohort who had IL-2 responses above the limit of detection (values below this limit were set at 0.1 to allow their visualisation on a logarithmic axis) for S, N and M peptides was higher in every case for positive controls than for unexposed. We used an internal positive control comprised of a mixture of anti-CD3 antibody plus Staphylococcal enterotoxin B (SEB) which confirmed that donor cells were capable of producing IL-2. Given that some studies have been carried out using IFN-γ responses to SARS-CoV-2, we determined the use of IFN-γ in Long COVID but this was confounded by high background. Although the median IFN-γ release T-cell responses to S, N and M peptides increased in the RT-qPCR-confirmed SARS-CoV-2 cohort of subjects, at D28, D90 and D180 relative to unexposed control (Figure 2a-d), differences were smaller than those seen for IL-2. Some PBMC from unexposed (pre-2019) donors had particularly high IL-2 responses to S, N and M which we attribute to T-cell cross-reactivity to circulating endemic human coronaviruses (Figure 2a-d). Indeed, sequence analysis confirmed that regions of S, N and M peptides are similar between SARS-CoV-2 and the endemic betacoronaviruses: HKU1 and OC43, (Figure S1).

Given that our data reflect that IL-2 responses show superiority over IFN-γ as a discriminator for past SARS-CoV-2 infection, we therefore chose to develop our assay in Long COVID patients based on IL-2 responses.

Although most of the Long COVID patients have now been vaccinated, we hereby only analysed data using samples taken prior to vaccination, to avoid confounding immune responses associated with vaccines. Based on the clinical history and temporal link, we expected that some patients were indeed infected with SARS-CoV-2, while others were likely to have been infected with other pathogens exhibiting overlapping symptoms with those of COVID-19. Due to this, we also stratified the Long COVID cohort into those who were seropositive for both anti-S and anti-N antibodies (21/72) and/or had a positive nasopharyngeal SARS-CoV-2 RT-qPCR swab (7/72), against those who were antibody negative and did not have a positive RT-qPCR test totalling 25 patients.

**Figure 3:**
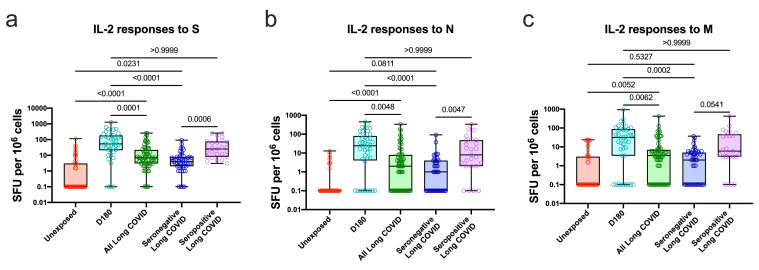
**Donors with diagnosed Long COVID show increased IL-2 responses to Spike, nucleocapsid and membrane peptides**. PBMCs were isolated from diagnosed Long COVID patients between 6-13 months after symptom onset. These PBMCs were stimulated with spike (A), nucleocapsid (B) or membrane (C) peptides. IL-2 responses were measured by fluorospot assay as spot forming units per million PBMCs. Unexposed (red), and PCR-positive samples at day 180 (cyan) are included from Figure 1. Results either show the entire Long COVID cohort (green) or those stratified by antibody serology as negative (blue) or positive (purple). Zero results are set as 0.1 to allow their inclusion on a log scale. Each condition was run in duplicate and an unstimulated control was subtracted to remove background cytokine production. L.O.D. = limit of detection. Significance calculated by Kruskal-Wallis ANOVA, with Dunn's multiple comparison test between every group.(For interpretation of the references to color in this figure legend, the reader is referred to the web version of this article.)

Our Long COVID cohort showed a range of IL-2 responses to S, M and N peptides, which is consistent with the cohort being comprised of some patients who genuinely had been infected and others who hadn't been infected with SARS-CoV-2. The analysis showed that all patients within the anti-S/anti-N seropositive group had detectable IL-2 T-cell responses to S peptides, and all but one individual also responded to the M and N peptides (Figure 3a-c). Overall, IL-2 secretion in response to spike, membrane or nucleocapsid peptide stimulation was not different as measured by Kruskal-Wallis ANOVA (*p*>0.999) between the Long COVID seropositive group and the D180 COVID-19 confirmed positive group. As expected, the Long COVID seronegative group was more varied, with some individuals having similar responses to M and N as our RT-qPCR COVID positive cohort, and others showing no detectable response (Figure 3a-c). Taken together, our findings show that SARS-CoV-2 specific IL-2 responses (summarised in Table 3) are sufficiently stronger in known positive cohorts, compared to unexposed controls, which would allow us to confidently identify other patients likely to have been infected with SARS-CoV-2 who lack a positive confirmatory SARS-CoV-2 RT-PCR or serological evidence of past infection.

**Table 3:** SARS-CoV-2 serology and IL-2 status of Long COVID patients.

	All Long COVID patients	Non-hospitalised	Hospitalised	SARS-CoV-2 antibody positive (negative)	IL-2 positivity (of which seronegative)
**Age categories**					
18–30	10	9	1	5 (5)	5 (1)
31–45	25	25	0	4 (21)	11 (9)
46–60	20	19	1	5 (15)	10 (5)
>60	17	7	10	6 (11)	13 (7)
**Gender**					
Female	44	35	9	10 (34)	23 (15)
Male	28	23	5	11 (17)	16 (7)

### Combining T cell IL-2 responses to N and M increases sensitivity for diagnosing previous SARS-CoV-2 infection

Although SARS-CoV-2 specific T cell IL-2 responses to S, N, and M were on average stronger in RT-qPCR confirmed positive patients than in unexposed donors, 25/54 (44%) unexposed donors had detectable responses to at least one peptide pool of S, N or M. As this may represent cross reactivity to other coronavirus epitopes, we therefore compared responses across two pools, to screen out low-level cross reactivity and increase the degree of confidence to detect true positive. As expected, this approach reduces the number of donors who show a positive response. For example, 20/54 (37%) unexposed donors respond to either M or N, but 7/54 (12.9%) respond to both.

**Figure 4:**
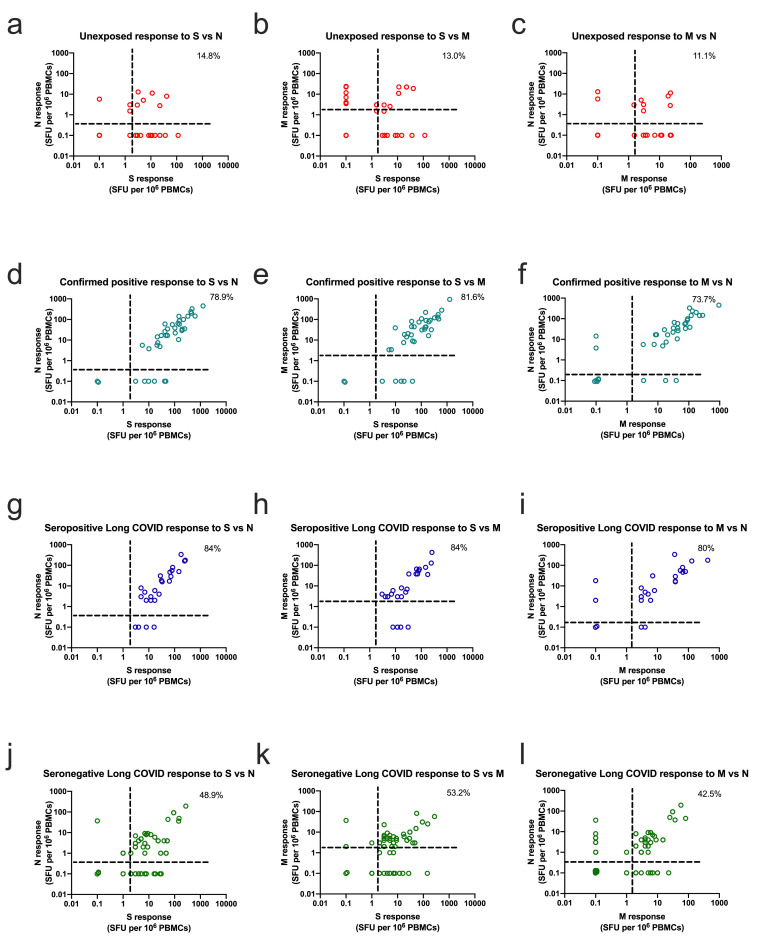
**Positive donors are likely to respond to multiple SARS-CoV-2 peptides while unexposed negative control donors do not**. Fluorospot results from Figure 1 plotted against each other. Dotted lines indicate results higher than the upper quartile for unexposed IL-2 responses to S, N, or M peptides.

We plotted individual patient and donor responses to each peptide pool and declared an individual positive if they had a response to S/M/N higher than any unexposed control, or if the individual responded to two or more open reading frames (ORFs) with both signals above the upper quartile of the unexposed control samples. By using this higher threshold we reduced positive responses for the unexposed control group (Figure 4a-c), down to 11–15% of the total cohort, but responses remained much higher for RT-qPCR confirmed positive patients at D180 (Figure 4d-f) at 73–81% of the cohort. The seropositive patients with Long COVID showed similarly strong responses to multiple SARS-CoV-2 specific peptides, at 80–84% positive (Figure 4g-i). Interestingly, 42–53% of Long COVID patients who were anti-S and anti-N seronegative showed clear positive T cell responses to two SARS-CoV-2 peptide pools (Figure 4j-l). Therefore, this assay is highly sensitive for the retrospective diagnosis of SARS-CoV-2 infection, detecting 73% (+/−13%) of known positive samples.

**Figure 5:**
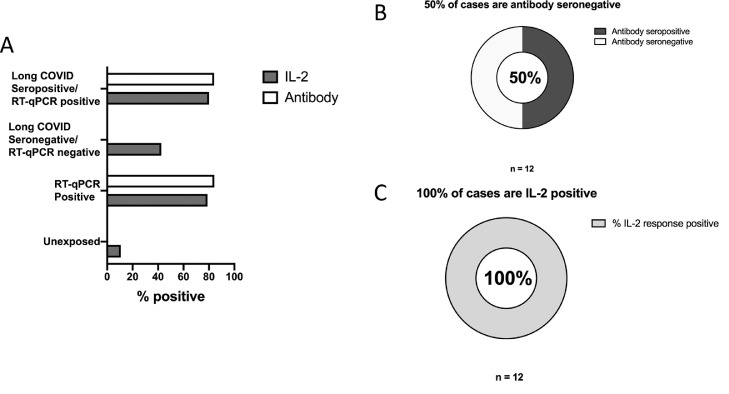
**IL-2 responses to N and M peptides are more sensitive to past SARS-CoV-2 infection than antibody serology**. A) Percentage of each cohort (unexposed, RT-qPCR positive at D180, Long COVID negative and long COVID positive) who had a positive IL-2 response to N and M peptides vs positive antibody responses. B) Percentage of COVID-19 hospitalised patients who were antibody positive for anti-S and anti-N antibodies. C) Percentage of COVID-19 hospitalised patients who were IL-2 positive for N and M peptides.

Given the increasing number of vaccinated individuals, we decided that using N and M responses to determine infection was the best approach to avoid confounding data from T cells induced by Spike-based vaccines. Indeed, samples from a selection of Long COVID patients after their first vaccine dose almost all showed increased IL-2 responses to spike (Figure S2). Excluding spike as a marker of infection therefore avoids this problem. Using N and M responses exclusively, our test identified (28/38) 74% of RT-qPCR known positives, at D180 post infection or (20/25) 80% of seropositive/RT-qPCR positive patients with Long COVID at least 6 months post infection (Figure 5a). This compares favourably to anti-spike, anti-N IgG serology, where (33/38) of known positives, at D180 and (21/25) of seropositive/RT-qPCR positive patients with Long COVID were positive. As we did not have blood serum samples for unexposed donors, we were unable to test this group for antibody positivity, however as our test currently detects responses in 11% of this cohort, we can estimate the sensitivity to be 88%. Additionally, our assay revealed that 42.5% of the patients within the Long COVID cohort had strong virus-specific T cell evidence for past infection with SARS-CoV-2, despite being seronegative (Figure 5a). Furthermore, of the 12 patients in our cohort who were hospitalised with COVID-19, 100% (12/12) were positive for SARS-CoV-2 IL-2 T cell responses while only 50% (6/12) were antibody seropositive (Figure 5b,c).

## Discussion

Long COVID or ‘post-acute sequelae of COVID-19’ (PASC) is likely driven by multiple pathophysiological mechanisms with a resultant plethora of symptoms following infection with SARS-CoV-2. As community testing was restricted to contacts of known cases and those in hospital until May 2020, many people who were asymptomatic to mildly symptomatic were not tested by nasopharyngeal swab RT-qPCR or antigen tests. Indeed within our cohort with Long COVID, 52/72 reported symptoms but were not hospitalised before this date and so were affected by these restrictions. The lack of SARS-CoV-2 RT-qPCR and antigen tests has compounded the diagnostic dilemma in patients now presenting with Long COVID. As such, serum IgG levels are used to determine past infection. However, IgG against nucleocapsid,^41^ spike receptor binding domain^20^ and S1/S2 units of spike protein^41^ wane beyond 6 months from the onset of symptoms, especially in those with asymptomatic or mild COVID-19 illness.^20^^,^^21^^,^^41^, ^42^, ^43^ The failure of a significant number of people to sustain high levels of antibody production after infection was also evident in our Long COVID cohort, albeit the numbers were limited, nonetheless the data was consistent with known literature (Figure S3). Going forward, this will prove to be a significant problem for retrospective diagnosis of SARS-CoV-2 infection and consequently diagnosing Long COVID. The loss of antibodies over time following coronaviruses closely related to SARS-CoV-2, i.e., SARS-CoV-1 and MERS infection, also suggests that antibodies have considerable limitations especially when used in isolation as diagnostic tools for past SARS-CoV-2 infection.^44^ The resultant diagnostic conundrum for those presenting with Long COVID has led to understandable frustrations for both clinicians and patients alike.

To address this urgent unmet clinical and scientific need, we tested whether a highly sensitive, dual-colour cytokine (IFN-γ and IL-2) T cell FluoroSpot assay could determine which patients presenting with symptoms of Long COVID had evidence of past infection with SARS-CoV-2. We found that 180 days after a positive RT-qPCR result for SARS-CoV-2 infection, patients' T cells produced IL-2 in response to stimulation with Spike, Nucleocapsid and Membrane protein peptides at significantly higher levels than in unexposed control group from 2014 to 2018 (Figure 1). This is consistent with published data that show decreasing antibody titres over time alongside detectable memory T cell responses.^30^^,^^45^

Confirming the efficacy of our assay, patients diagnosed with Long COVID who were either antibody positive or who had a positive RT-qPCR result after nasopharyngeal swab were highly likely to respond to SARS-CoV-2 peptide stimulation with IL-2 release (Figure 3). To improve stringency of our assay we excluded anti-Spike responses which could be caused by vaccination (Figure S2) and used responses to M and N as criteria for a positive result (Figure 4). Excluding Spike responses did not significantly reduce the number of identified patients, as most patients who responded to Spike also responded to both N and M, consistent with the findings of others.^32^^,^^33^^,^^46^ Using this approach, we were able to identify that 42.5% (+/−11%) of seronegative patients with symptoms in keeping with Long COVID in our cohort had indeed been infected with SARS-CoV-2 at some point in their illness trajectory (Figure 5). We cannot rule out the possibility that some people were infected with SARS-CoV-2 but did not develop detectable memory immune responses (either antibodies or T cells). However, as our Long COVID cohort were recruited based on patient-reported symptoms, we expect that some patients were genuinely never infected, and are experiencing symptoms due to another condition.

Our findings are consistent with other coronaviruses where cellular immunity is also important.^47^ T cell responses were detectable >10 years after infection with SARS-CoV-1 despite undetectable IgG in 2/23 patients,^42^^,^^48^, ^49^, ^50^, ^51^ suggesting that with the passage of time, T cell-based assays such as our fluorospot approach are more effective and sensitive than antibody serology. There is also proof of concept in the use of T cell-based ELISpot assays for diagnosis of latent *Mycobacterium Tuberculosis* (using antigens ESAT-6 and CFP-10).^28^ Indeed, T-SPOT.COVID from Oxford Immunotec uses IFN-γ release as a measure of past SARS-CoV-2 infection (https://www.tspotcovid.com/), which has been used as a measure of historical SARS-CoV-2 infection by some^52^ and proposed as a diagnostic tool by others^34^ but never investigated in the context of Long COVID. We concur with their analysis showing that T cell responses do not wane as quickly as antibody serology. However, our data suggest that IL-2 is a superior discriminator to IFN-γ, as the latter exhibits higher results in unexposed individuals (Figure 1), hence the concern it could lead to higher false positive rates. Exactly why IL-2 is a better discriminator than IFN-γ can be answered by a potential bystander effect, where high levels of IFN-γ are produced in unstimulated cells at D28 and D90 post infection.^35^ Separation studies suggest IFN-γ is produced by CD8+ T cells, suggesting transient dysregulation of these cells.^38^ This background IFN-γ reduces sensitivity for the fluorospot assay, making it a less useful biomarker of past SARS-CoV-2 infection.

In our Long COVID cohort, based on serology test, 21/72 patients had been infected with SARS-CoV-2; however, using the T cell assay, interestingly and more importantly, this enabled us to identify an additional 22 patients, therefore, doubling the number of patients we believe had previously been infected with SARS-CoV-2. As this assay would not be any more invasive than antibody serology as blood is used for both tests, and the T cell assay can be performed using only 10^7^ PBMCs, or around 10 ml of blood. It would be interesting in future work with a larger cohort to determine whether there is any correlation between likelihood of past infection with certain symptoms to ideally narrow the range of symptoms associated with Long COVID. Other attempts so far have found that fatigue is associated with female gender, pre-existing lung disease, severity of acute illness and increased convalescent antibody titres.^48^ Our data does not show correlation between any particular symptoms or resolution of symptoms with IL-2 responses.

The T cell reactivity noted with all 3 peptide pools (S/N/M) in the unexposed donors is probably due to cross-reactivity with other betacoronaviruses, which has been previously reported,^29^^,^^53^, ^54^, ^55^, ^56^, ^57^ and confirmed here using basic sequence alignment (Figure S1), which found that 37.4%/35.7%, 40.8%/37.1% and 42%/37% sequence homology for SARS-CoV-2 to S, M and Nc for OC43/HKU1 respectively. Indeed, 3/54 unexposed donors had strong responses to S, N and M which were too high to disregard as background production. We used pools of peptides to stimulate PBMCs which covered the entire length of the SARS-CoV-2 open reading frames for S/N/M. There is scope to modify the pool of peptides used, in-order to further reduce the false positive rate. We will start by excluding peptides that we identified by sequence alignment of S/N/M from SARS-CoV-2 with the circulating human coronaviruses and SARS-CoV-1 (Figure S1). This is likely to be a key issue as the SARS-CoV-2 T cell reactive unexposed individuals within the negative control group were likely infected with one or more of the four human circulating endemic coronaviruses that are known to cause the common cold. It is less likely that our Long COVID cohort have been infected recently with other coronaviruses, as infections with all respiratory viruses dropped during 2020 due to non-pharmacological interventions including social distancing and wearing of face masks, directed against SARS-CoV-2.^49^^,^^58^ This will however become an issue for retrospective diagnosis of SARS-CoV-2 infection using T cell-based assays as countries end restrictions on social interactions, which will likely see resurfacing and the spread of circulating endemic coronaviruses. Reducing cross reactivity will therefore be paramount to attain higher accuracy in the future. In addition to this, our cohorts were infected with SARS-CoV-2 in April–October 2020, allowing us to use the Wuhan sequence of peptides. Future work will need to include peptides covering other vairants as well as variants which have yet to emerge. Use of peptides from non-structural proteins to detect T cell responses to SARS-CoV-2 has been done in other settings,^33^^,^^59^^,^^60^ however, these are less immunogenic.

Although no single test is likely to be the panacea for diagnosis of this complex heterogeneous disease, the development of a diagnostic assay to previous SARS-CoV-2 infections that circumvent the limitations of serological based assays would be beneficial to both Long COVID patients and clinicians in planning future treatments. Our assay has revealed that 42.5% of patients with symptoms suggestive of Long COVID from the initial phase of the pandemic who had been missed out by SARS-CoV-2 serological assays, have indeed been infected with SARS-CoV-2. Based on our findings we propose that IL-2 production, in addition to antibody assays, will allow for more sensitive detection of previous SARS-CoV-2 infections. The assay can potentially be adapted to a simpler whole blood peptide stimulation assay with IL-2 ELISA readout, thereby attaining high-throughput advantages and an easier to implement clinical diagnostic assay.

## Contributors

Conceptualisation, MRW, NS; Methodology, MRW, NS, BAK; Investigation & Data Collection, BAK, EYL, LM, RD, MRW, NS; Supervision – NJM, PJL, MRW, NS; Writing – Original Draft, BAK, MRW, NS; Writing – Review & Editing, BAK, EYL, LM, PL, RD, JB, KGCS, JS, NJM, PJL, MRW and NS; Collection and processing of blood samples (D 28, 90, 180) – NIHR BioResource.

## NIHR BioResource

John Allison, Heather Biggs, John Bradley, Helen Butcher, Daniela Caputo, Matt Chandler, Debbie Clapham-Riley, Patrick Chinnery, Anne-Maree Dean, Eleanor Dewhurst, Christian Fernandez, Anita Furlong, Anne George, Barbara Graves, Jennifer Gray, Sabine Hein, Tasmin Ivers, Mary Kasanicki, Nathalie Kingston, Emma Le Gresley, Rachel Linger, Sarah Meloy, Alexei Moulton, Francesca Muldoon, Nigel Ovington, Roxana Paraschiv, Sofia Papadia, Isabel Phelan, Christopher Penkett, Venkatesh Ranganath, Jennifer Sambrook, Katherine Schon, Hannah Stark, Kathleen E Stirrups, Paul Townsend, Julie von Ziegenweidt, Neil Walker, Jennifer Webster.

## Data sharing statement

Deidentified data will be shared by authors upon request, which can include patient responses, consent forms.

## Declaration of interests

The authors declare no competing interests

## Appendix Supplementary materials

Image, application 1
