# Supplementary material for: Evidence of previous SARS-CoV-2 infection in seronegative patients with long COVID
Source: eBioMedicine. 2022 Jun 27;81:104129. doi: 10.1016/j.ebiom.2022.104129 (PMC9235296; doi:10.1016/j.ebiom.2022.104129)
Supplement: Supplementary file 1 [file mmc1.docx]

Spike alignment


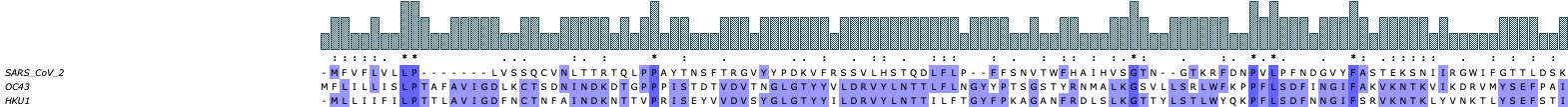

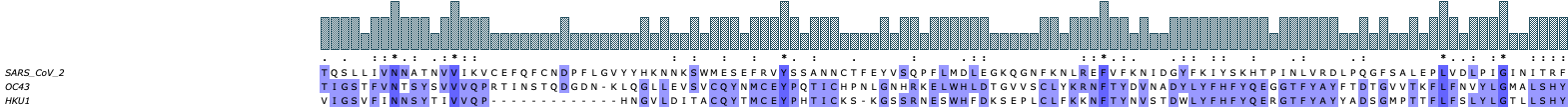

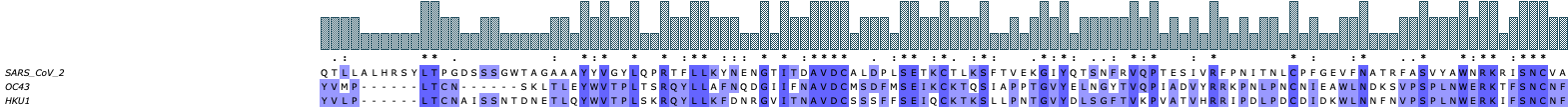

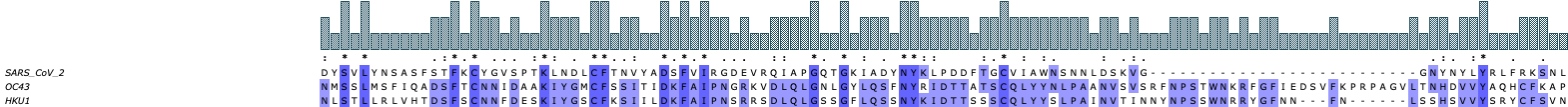

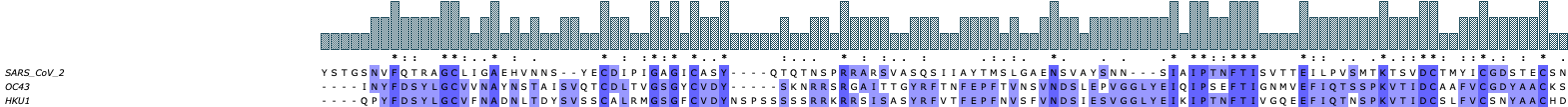

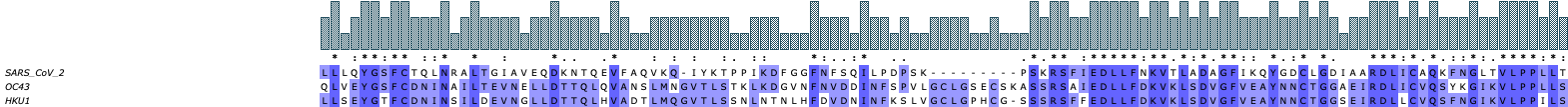

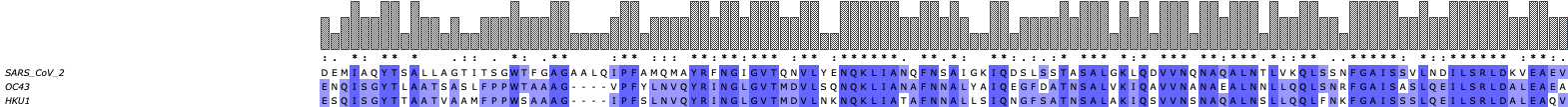

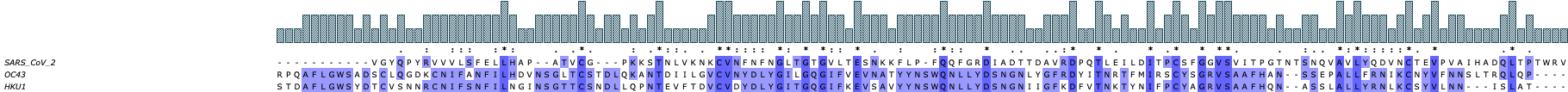

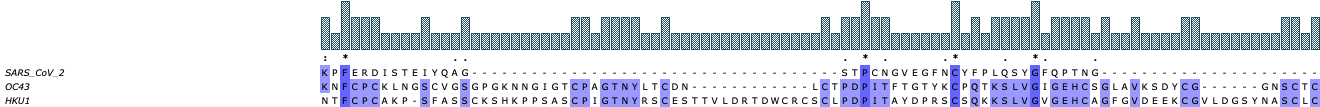

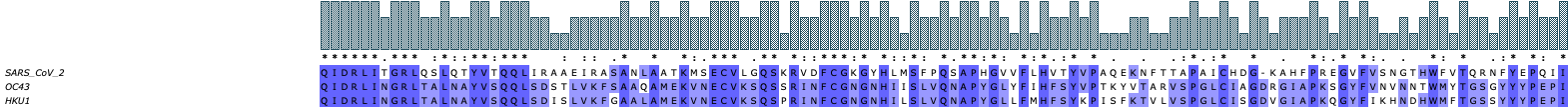

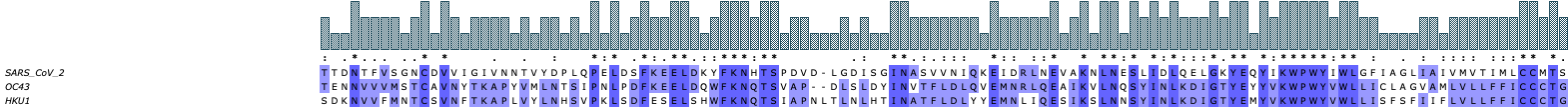

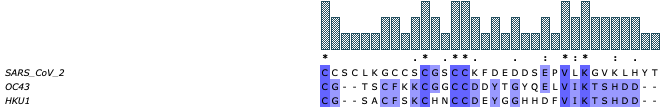


Nucleocapsid alignment


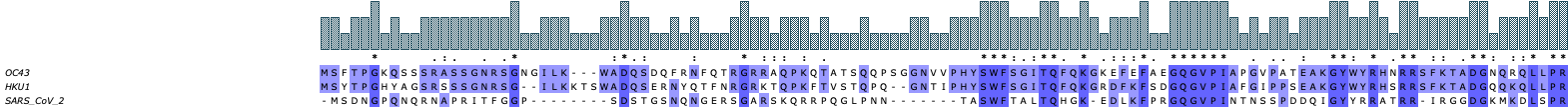

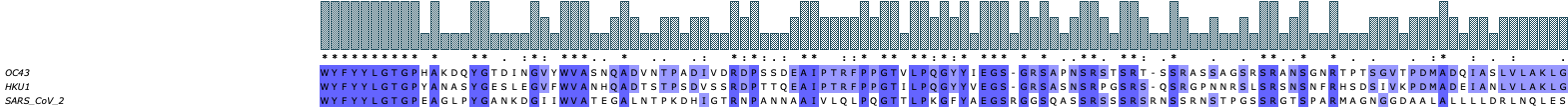

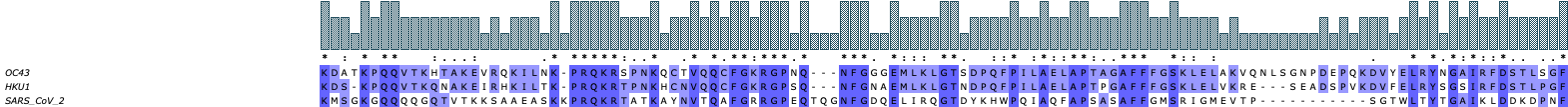

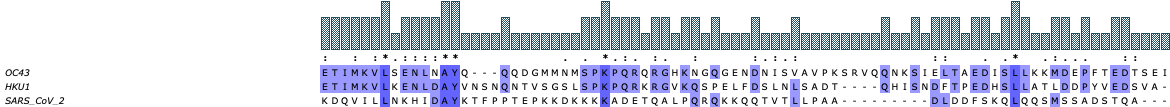


Membrane alignment


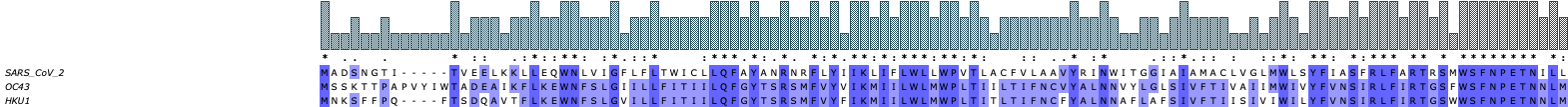

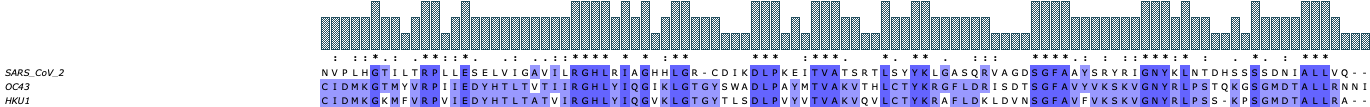


**Figure S1: Alignment of peptide sequences from spike, nucleocapsid and membrane proteins shows areas of overlap for each protein.** Alignments performed using clustal omega and visualised by UGENE.

**Figure S2: Vaccination increases IL-2 responses to Spike but not nucleocapsid or membrane peptides.** PBMCs were isolated from 17 donors with diagnosed long COVID pre- and post-vaccination. These PBMCs were stimulated with spike, nucleocapsid or membrane peptides and IL-2 responses were measured by fluorospot assay as spot forming units per million PBMCs. Each condition was run in duplicate and an unstimulated control was subtracted to remove background cytokine production. P<0.01 and ns= not significant by Wilcoxon Signed-Rank Test.

**Figure S3: Spike antibodies are confounded by vaccination while nucleocapsid antibodies wane quickly.** Antibody levels were quantified from 6 donors before and after vaccination using multiplex particle-based flow cytometry for A) spike (not significant by Wilcoxon Signed-Rank) or B) nucleocapsid antibodies (p<0.05 by Wilcoxon Signed-Rank).
